# Biochemical and genetic examination of two aminotransferases from the hyperthermophilic archaeon *Thermococcus kodakarensis*

**DOI:** 10.3389/fmicb.2023.1126218

**Published:** 2023-02-20

**Authors:** Yu Su, Yuta Michimori, Haruyuki Atomi

**Affiliations:** ^1^Department of Synthetic Chemistry and Biological Chemistry, Graduate School of Engineering, Kyoto University, Kyoto, Japan; ^2^Integrated Research Center for Carbon Negative Science, Kyoto University, Kyoto, Japan

**Keywords:** Archaea, aminotransferase, metabolism, enzyme, genetics

## Abstract

The hyperthermophilic archaeon *Thermococcus kodakarensis* utilizes amino acids as a carbon and energy source. Multiple aminotransferases, along with glutamate dehydrogenase, are presumed to be involved in the catabolic conversion of amino acids. *T. kodakarensis* harbors seven Class I aminotransferase homologs on its genome. Here we examined the biochemical properties and physiological roles of two Class I aminotransferases. The TK0548 protein was produced in *Escherichia coli* and the TK2268 protein in *T. kodakarensis*. Purified TK0548 protein preferred Phe, Trp, Tyr, and His, and to a lower extent, Leu, Met and Glu. The TK2268 protein preferred Glu and Asp, with lower activities toward Cys, Leu, Ala, Met and Tyr. Both proteins recognized 2-oxoglutarate as the amino acceptor. The TK0548 protein exhibited the highest *k*_cat_/*K*_m_ value toward Phe, followed by Trp, Tyr, and His. The TK2268 protein exhibited highest *k*_cat_/*K*_m_ values for Glu and Asp. The TK0548 and TK2268 genes were individually disrupted, and both disruption strains displayed a retardation in growth on a minimal amino acid medium, suggesting their involvement in amino acid metabolism. Activities in the cell-free extracts of the disruption strains and the host strain were examined. The results suggested that the TK0548 protein contributes to the conversion of Trp, Tyr and His, and the TK2268 protein to that of Asp and His. Although other aminotransferases seem to contribute to the transamination of Phe, Trp, Tyr, Asp, and Glu, our results suggest that the TK0548 protein is responsible for the majority of aminotransferase activity toward His in *T. kodakarensis*. The genetic examination carried out in this study provides insight into the contributions of the two aminotransferases toward specific amino acids *in vivo*, an aspect which had not been thoroughly considered thus far.

## Introduction

Aminotransferases, or transaminases, catalyze the reversible transfer of an amino group from a donor to a keto group of an acceptor compound (Toyokawa et al., 2021; Koper et al., 2022). The reaction is dependent on pyridoxal 5′-phosphate (PLP). Many aminotransferases recognize the α-amino group of α-amino acids and the keto group of 2-oxoacids, but some recognize the amino group on side chains (Koszelewski et al., 2010; Malik et al., 2012; Zheng et al., 2018) or substrates other than α-amino acids (Gomm and O'Reilly, 2018; Kelly et al., 2020). The enzymes play a pivotal role in the carbon and nitrogen metabolism in a wide range of organisms. Aminotransferases are a focus of attention due to their involvement in a variety of diseases (Kunutsor et al., 2013; Montioli et al., 2021) and are also attractive enzymes for their use in biocatalysis (Malik et al., 2012; Gomm and O'Reilly, 2018; Kelly et al., 2020).

Aminotransferases can be divided into four classes (Class I to IV) based on their primary structure (Mehta et al., 1993). On the other hand, PLP-dependent enzymes can be classified by their folds (Fold type I to VII; Jansonius, 1998; Schneider et al., 2000; Koper et al., 2022). Aminotransferase Class I, II, and IV share a common ancestor with a Fold type I whereas Class III aminotransferases are members of Fold type IV PLP enzymes (Mehta et al., 1993). Class I enzymes constitute the majority of aminotransferases, including aromatic aminotransferases, aspartate aminotransferases, and alanine aminotransferases. Class II includes enzymes that can recognize amino groups other than the α-amino group of amino acids and utilize substrates such as γ-aminobutyric acid, or GABA, and ornithine. Class III enzymes with the distinct fold type IV utilize D-amino acids and branched-chain amino acids. Class IV enzymes utilize Ser, *O*-phosphoserine or Asp as the amino donor (Mehta et al., 1993; Mehta and Christen, 2000; Koper et al., 2022).

Thermococcales consists of three genera, *Pyrococcus*, *Thermococcus*, and *Palaeococcus*, and members readily utilize amino acids and peptides as a carbon and energy source for growth (Zillig et al., 1983; Fiala and Stetter, 1986; Takai et al., 2000). Different enzymes and pathways involved in amino acid catabolism and biosynthesis have been studied in these organisms. In particular, the enzymatic properties of a wide range of aminotransferases from *Pyrococcus furiosus* (Andreotti et al., 1995; Ward et al., 2000, 2002), *Pyrococcus horikoshii* (Matsui et al., 2000; Ura et al., 2001; Okada et al., 2012, 2014), *Thermococcus litoralis* (Andreotti et al., 1994; Sakuraba et al., 2004, 2008), *Thermococcus* sp. CKU-1 (Uchida et al., 2014) and *Thermococcus kodakarensis* (Kanai et al., 2015; Zheng et al., 2018) have been reported. Our group has been examining amino acid metabolism in *T. kodakarensis* (Shikata et al., 2007; Yokooji et al., 2013; Awano et al., 2014). In addition to the genome sequence (Fukui et al., 2005), a versatile genetic system (Sato et al., 2003, 2005; Matsumi et al., 2007; Santangelo et al., 2008, 2010) allows us to evaluate the physiological roles of individual genes in this organism.

A biochemical and genetic examination of enzymes/genes involved in Glu metabolism in *T. kodakarensis* confirmed the presence of glutamate dehydrogenase (TK1431), catalyzing an NADP-dependent interconversion between Glu and 2-oxoglutarate (Yokooji et al., 2013). The functions of 2-oxoglutarate:ferredoxin oxidoreductase (TK1123-TK1125, TK1131) and ADP-forming succinyl-CoA synthetase (TK1880, TK0943; Shikata et al., 2007) were also identified, constituting a route from Glu to succinate. The latter two reactions lead to the generation of reduced ferredoxin and ATP coupled to 2-oxoacid degradation, providing reducing equivalents and chemical energy (Yokooji et al., 2013). The presence of four 2-oxoacid:ferredoxin oxidoreductases and five ADP-forming acyl-CoA synthetases in *T. kodakarensis* suggest that other amino acids are catabolized through similar reactions. Based on the substrate specificities of the acyl-CoA synthetases (Mai and Adams, 1996; Glasemacher et al., 1997; Musfeldt et al., 1999; Shikata et al., 2007; Awano et al., 2014), it was suggested that the following amino acids are subject to catabolism; Ala, Val, Leu, Ile, Met, Phe, Tyr, Trp, Glu/Gln, Cys, and His (Awano et al., 2014). However, dehydrogenases acting on amino acids other than Glu such as aspartate dehydrogenase and alanine dehydrogenase have been genetically confirmed to be absent in *T. kodakarensis* (Yokooji et al., 2013). This implies that 2-oxoacid generation from amino acids relies on aminotransferases. In order to better understand which amino acids are subject to catabolic degradation and which aminotransferases are involved, here we carried out a biochemical and genetic examination on two aminotransferases in *T. kodakarensis* encoded by TK0548 and TK2268.

## Results

### Multiple groups of aminotransferase homologs in Thermococcales

**Figure 1:**
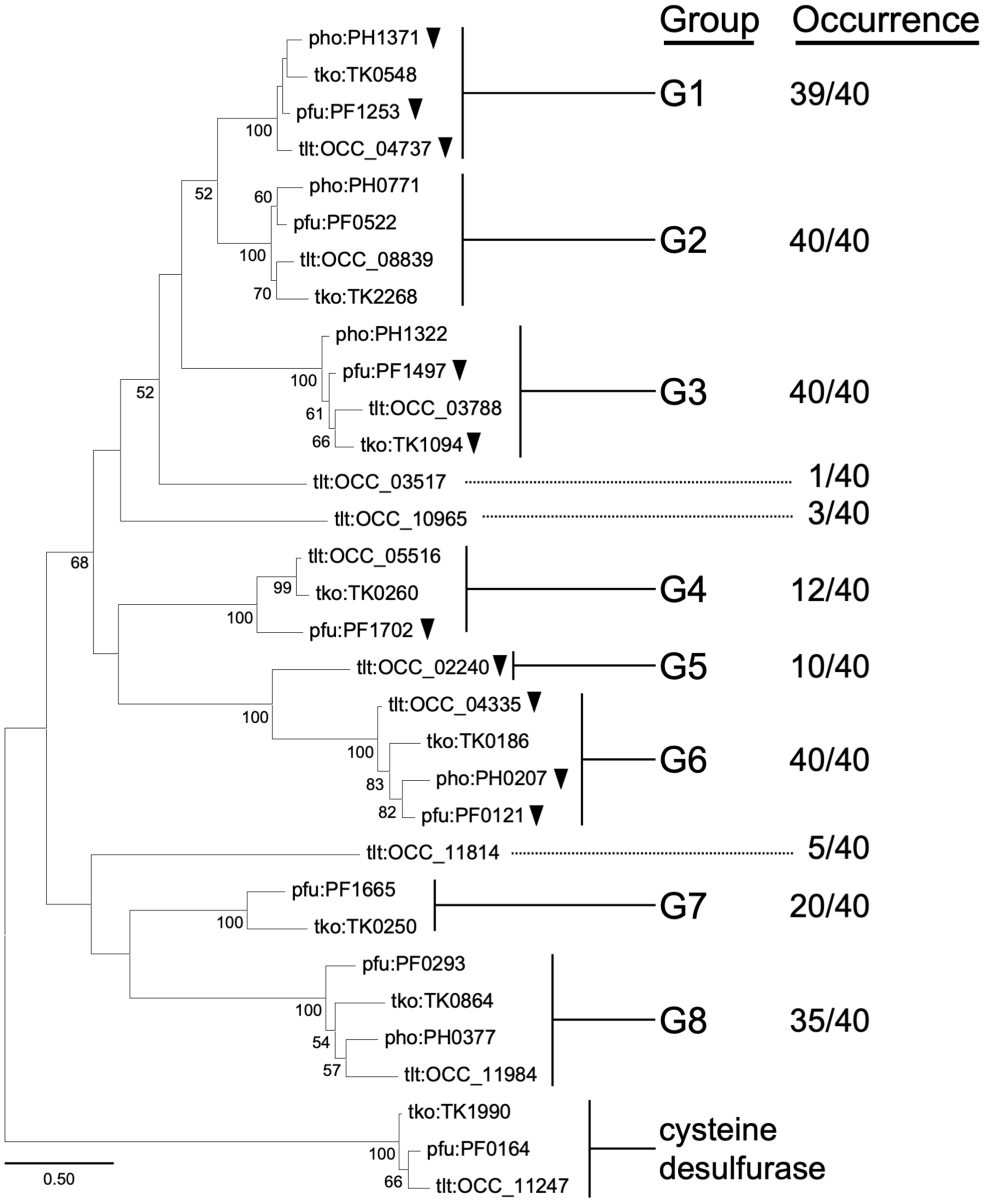
Phylogenetic analysis of Class I aminotransferases from selected species of Thermococcales. Amino acid sequences were collected from *T. kodakarensis*, *T. litoralis*, *P. furiosus*, and *P. horikoshii*. Homologs of cysteine desulfurase (TK1990) were added into the dataset as an outgroup. The sequences were aligned using MUSCLE algorithm (Edgar, 2004). The phylogenetic analysis was performed using the Maximum Likelihood method and JTT matrix-based model (Jones et al., 1992). The tree with the highest log likelihood (−9712.68) is shown. The percentage of trees in which the associated taxa clustered together is shown next to the branches. Bootstrap values above 50 are shown. Initial tree(s) for the heuristic search were obtained automatically by applying Neighbor-Join and BioNJ algorithms to a matrix of pairwise distances estimated using the JTT model, then selecting the topology with superior log likelihood value. The tree is drawn to scale, with branch lengths measured in the number of substitutions per site. This analysis involved 32 amino acid sequences. All positions containing gaps and missing data were eliminated (complete deletion option). There were a total of 242 positions in the final dataset. Evolutionary analyses were conducted in MEGA11 (Tamura et al., 2021). Homologs that have been characterized genetically or biochemically are indicated with black arrowheads. Information on the groups or occurrence is indicated in the text.

Figure 1 shows a phylogenetic tree of Class I aminotransferase homologs from four selected Thermococcales species, *T. kodakarensis*, *P. furiosus*, *P. horikoshii*, and *T. litoralis*, from which multiple aminotransferases have been experimentally studied. A clade including TK1990, a representative of a Class IV protein encoding cysteine desulfurase (Hidese et al., 2014), was used as the outgroup. There are eight groups (G1–G8) in which homologs occur in ten or more genomes of the 40 Thermococcales species examined. Among the eight groups, members of G1 (39 genomes among the 40 genomes examined harbor homologs; 39/40), G2 (40/40), G3 (40/40), G6 (40/40), and G8 (35/40) are present in most or all of the Thermococcales genomes used in our analysis. Members of G4 (12/40), G5 (10/40), and G7 (20/40) are less distributed. *T. litoralis* harbors a higher number of Class I aminotransferase homologs compared to the other three species. Homologs of OCC_03517 are not found on any of the other Thermococcales genomes, but homologs of OCC_10965 (3/40), OCC_02240 (10/40, G5), and OCC_11814 (5/40) can be identified in other species. A detailed phylogenetic tree based on sequences of all Class I aminotransferase homologs from Thermococcales species is shown in Supplementary Figure S1.

We considered the physiological roles of these groups of aminotransferases by first taking into account their gene location and previous biochemical studies on aminotransferases from members of Thermococcales. In terms of gene location, we observed that TK0250 (G7) is included within the His biosynthesis gene cluster (TK0242-TK0251) in *T. kodakarensis*. We also observed a complete co-occurrence between the TK0250 homologs and the His biosynthesis operon in Thermococcales members (Supplementary Table S1). A similar situation was observed for TK0260 (G4). The gene is situated in a gene cluster (TK0259-TK0261) involved in the biosynthesis of Phe/Tyr in *T. kodakarensis*, and co-occurrence is observed between TK0260 homologs and the gene cluster in Thermococcales species (Supplementary Table S2). These observations suggest that the physiological roles of members of G4 and G7 are related to Phe/Tyr and His biosynthesis, respectively. The members of G8 from Thermococcales have not been characterized, but display 30% identity to Thr decarboxylase (encoded by MM2060) from *Methanosarcina mazei* (Tavares et al., 2018, 2019) and are clustered with genes related to cobalamin salvage. By contrast, members of G1, G2, G3, G5, and G6 did not show a tendency to be included in a particular biosynthesis operon or gene cluster.

Members of G1, G2, G3, and G6 are widely distributed among Thermococcales species, and a number of members have been biochemically characterized. In particular, members of G1 and G6 have been relatively well studied. In the case of G1, the structure of the PH1371 protein from *P. horikoshii* has been elucidated, and the amino donors most recognized were Tyr, Phe, Glu, His, and Trp (Matsui et al., 2000). The PF1253 protein from *P. furiosus*, referred to as AroAT II, displayed preference to Phe, Tyr and Trp, with highest *k*_cat_/*K*_m_ toward Phe (Ward et al., 2002). The OCC_04737 protein from *T. litoralis*, referred to as ArAT II, also displayed similar properties, preferring Phe, Tyr, and Trp (Andreotti et al., 1994). In the case of G6, the PF0121 protein from *P. furiosus*, referred to as ArAT or AroAT I, displayed significant activity toward Phe, Trp, and Tyr (Andreotti et al., 1995). The OCC_04335 protein from *T. litoralis*, referred to as ArAT I, also recognized Phe, Tyr, and Trp (Andreotti et al., 1994). The PH0207 protein displays kynurenine aminotransferase activity which is involved in the degradation of Trp (Okada et al., 2012), and the crystal structure has been determined (Chon et al., 2005; Okada et al., 2014). Concerning other groups, the PF1497 protein from *P. furiosus* (G3), referred to as AlaAT, displays highest activity with Ala. The enzyme did not display activity toward Phe and Tyr (Ward et al., 2000). A genetic analysis on TK1094 from *T. kodakarensis* (G3) suggested a role in the conversion between pyruvate and Ala (Kanai et al., 2015). The PF1702 protein (G4), referred to as AspAT, displays highest activity toward Asp and Glu, with low activities to a broad range of amino acids (Ward et al., 2002).

We carried out further analyses on TK2268 and TK0548. The TK2268 protein is a member of G2, which does not have any member that has been experimentally characterized. Determining its biochemical properties would contribute to our understanding of amino acid metabolism in *T. kodakarensis*. We also examined the TK0548 protein, the most closely related protein to the TK2268 protein (45.4% identical). None of the G1 and G2 genes have been examined genetically.

### Expression of the TK0548 and TK2268 genes and purification of the recombinant proteins

**Figure 2:**
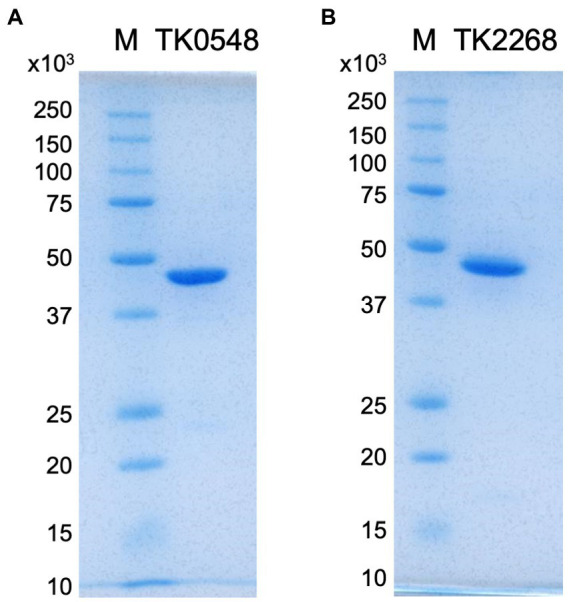
SDS-PAGE analyses of the purified TK0548 **(A)** and TK2268 **(B)** recombinant proteins. Two micrograms of purified TK0548 and TK2268 recombinant proteins were applied to each gel. Gels were stained with Coomassie Brilliant Blue. *M* indicates molecular weight markers.

In order to obtain recombinant proteins, the TK0548 and TK2268 genes were expressed in *Escherichia coli*. In the case of TK0548, soluble protein was obtained, and the recombinant TK0548 protein was purified through heat treatment, anion exchange chromatography and gel-filtration chromatography. In the case of TK2268, expression in *E. coli* resulted in the formation of inclusion bodies. The gene was thus expressed in the native host *T. kodakarensis* under the control of a strong constitutive promoter of the cell surface glycoprotein gene (*csg*, TK0895; Yokooji et al., 2009). Sequences to incorporate a His_6_-tag on the C-terminus of the TK2268 protein were introduced. The soluble TK2268 protein obtained in the cell extract of *T. kodakarensis* cells was purified using a nickel affinity column and gel filtration chromatography. Both proteins were subjected to SDS-PAGE, confirming their apparent homogeneities (Figure 2).

### Oligomeric state of the TK0548 and TK2268 proteins

The molecular masses of the purified, recombinant TK0548 and TK2268 proteins were examined with gel-filtration chromatography. The estimated molecular mass of the TK0548 protein was 85 kDa, and considering that the calculated molecular mass of the monomer was 43,608 Da, this suggested that the TK0548 protein was a dimer. In the case of the TK2268 protein, we consistently observed two peaks, corresponding to estimated molecular masses of 98 kDa and 327 kDa. As the calculated molecular mass of the monomer was 45,116 Da, the result suggested that the TK2268 protein forms a dimer unit, which may then further assemble to form an octamer.

### The TK0548 and TK2268 proteins catalyze transamination in a PLP-dependent manner

The purified TK0548 and TK2268 proteins were examined for aminotransferase activity. We used Glu (10 mM) as the amino group donor and pyruvate (10 mM) as the amino acceptor. We observed aminotransferase activity in both proteins. When PLP was omitted from the reaction, we observed a partial decrease in activity in both cases (TK0548: 13% decrease, TK2268: 50% decrease) compared to those observed with the addition of PLP, suggesting that the TK0548 and TK2268 proteins are PLP-dependent aminotransferases. The only partial decrease in activity is most likely due to PLP bound to the proteins when they were produced in their respective host cells, *E. coli* (TK0548) and *T. kodakarensis* (TK2268). To further support the necessity of PLP for the reactions, we added hydroxylamine, a PLP inhibitor (Kito et al., 1978) to the enzymes in the absence of supplemental PLP. In this case, we observed a further 93% decrease in activity of the TK0548 protein and a 78% decrease in activity of the TK2268 protein with the addition of hydroxylamine (Supplementary Figure S2).

### Transamination with varying amino donor and acceptor compounds

**Figure 3:**
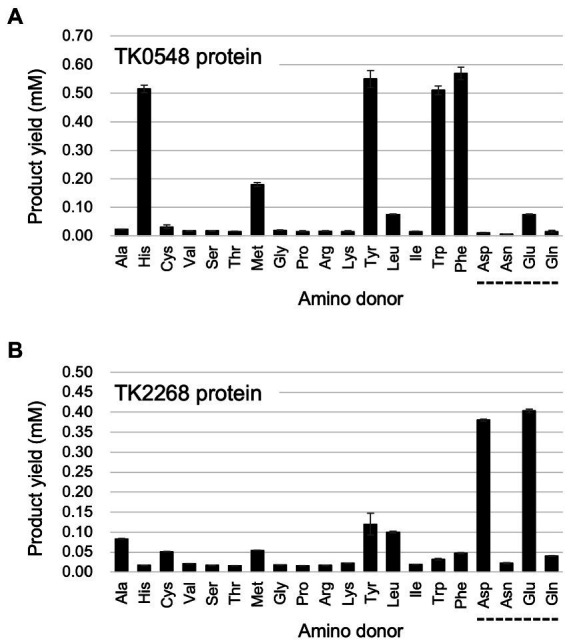
Examination of the amino donors recognized by the recombinant TK0548 **(A)** and TK2268 **(B)** proteins. Each protein was incubated for 15 min at 80°C with the indicated amino acids and 2-oxoglutarate and the generation of Glu was examined. The amino acids indicated with a dotted line below them were examined using pyruvate as the amino acceptor, and the generation of Ala was examined. Amino donors and acceptors were added at a concentration of 10 mM, with the exception of Tyr (6 mM). The results are the means of three independent assays and error bars indicate standard deviations.

We first carried out an initial screening to identify the amino acids recognized by the TK0548 and TK2268 proteins. 2-Oxoglutarate or pyruvate was used as the amino acceptor, and the production of Glu or Ala, respectively, was examined after 15 min. The substrates were present in the reaction mixture at a concentration of 10 mM. As shown in Figure 3A, the TK0548 protein utilized Phe, Tyr, His, and Trp as an amino donor, as well as Met, Glu, and Leu to a lower extent. In the case of the TK2268 protein, Asp and Glu were utilized, along with Tyr, Leu, Ala, Met, and Cys (Figure 3B).

**Figure 4:**
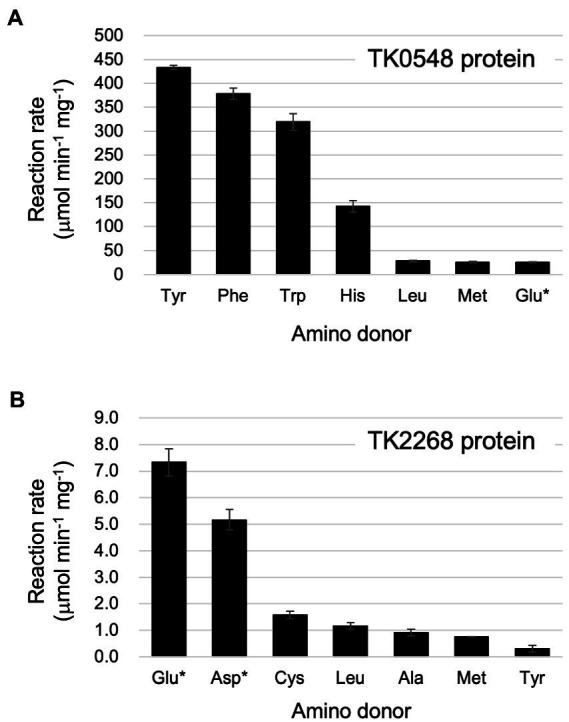
Reaction rates of the recombinant TK0548 **(A)** and TK2268 **(B)** proteins with various amino donors. Each protein was incubated for varying periods of time at 80°C with the indicated amino acids and 2-oxoglutarate or pyruvate (indicated with asterisks), and the reaction rates of Glu or Ala production, respectively, were calculated. Amino donors and acceptors were added at a concentration of 10 mM (Tyr: 6 mM). The results are the means of three independent assays and error bars indicate standard deviations.

Focusing on the amino acids that were recognized by the proteins, we examined enzyme activity. The amino acids and amino acceptors were constant at 10 mM. As shown in Figure 4A, the TK0548 protein exhibited highest activity toward Tyr, followed by Phe, Trp, and His. Activity toward Leu, Met, and Glu were much lower. On the other hand, the TK2268 protein displayed highest activity toward Glu, followed by Asp. Lower levels of activity were observed using Cys, Leu, Ala, Met, and Tyr (Figure 4B).

### Substrate specificity of the TK0548 and TK2268 proteins

Kinetic analyses were performed on the substrates that resulted in relatively high levels of activity. For the TK0548 protein, activities were first measured for varying concentrations of pyruvate or 2-oxoglutarate in the presence of 10 mM Phe. As shown in Table 1, the TK0548 protein exhibited a higher *V*_max_ and lower *K*_m_ toward 2-oxoglutarate when compared to pyruvate. The *k*_cat_/*K*_m_ value toward 2-oxoglutarate was over 70-fold higher than that for pyruvate. We next examined activity with varying concentrations of Phe, Tyr, Trp, His, and Met in the presence of 10 mM 2-oxoglutarate (Table 1). The highest *k*_cat_/*K*_m_ value was observed with Phe, with high values also observed for Tyr and Trp. A lower *k*_cat_/*K*_m_ value was observed with His, and that for Met was extremely low. The results suggested that the TK0548 protein mainly utilizes the aromatic amino acids Phe, Tyr, and Trp as substrates, with His also a possible substrate.

**Table 1:** Kinetic parameters of the aminotransferase reaction catalyzed by the TK0548 protein.

Substrate		*V*_max_ (μmol min^−1^ mg^−1^)	*K*_m_ (mM)	*k*_cat_ (s^−1^)	*k*_cat_/*K*_m_ (mM^−1^ s^−1^)
Amino donor	Amino acceptor				
*Phenylalanine*	2-Oxoglutarate^a^	483 ± 10	0.67 ± 0.06	351 ± 10	524
*Tyrosine*		419 ± 18	1.4 ± 0.2	305 ± 19	218
*Tryptophan*		359 ± 10	1.1 ± 0.1	261 ± 10	237
*Histidine*		179 ± 6	4.4 ± 0.4	130 ± 6	29.6
*Methionine*		169 ± 14	64 ± 9	123 ± 14	1.92
Phenylalanine^a^	*2-Oxoglutarate*	334 ± 10	1.5 ± 0.2	243 ± 10	162
	*Pyruvate*	137 ± 7	48 ± 5	100 ± 7	2.07

The kinetic parameters are those for the substrates indicated in italic. ^a^Concentration fixed at 10 mM.

Concerning the TK2268 protein, Leu (50 mM) was used as the amino donor to measure activity with varying concentrations of pyruvate and 2-oxoglutarate. The *k*_cat_/*K*_m_ value toward 2-oxoglutarate was higher than that toward pyruvate (Table 2). This was mainly due to differences in the *K*_m_ value, as their *V*_max_ values were comparable. As the product of the aminotransferase reaction with 2-oxoglutarate would not be possible with Glu as the amino donor, 10 mM pyruvate was used as the amino acceptor to examine the activities with varying concentrations of Glu, Asp, and Leu. As a result, relatively high *k*_cat_/*K*_m_ values were observed for both Asp and Glu compared to Leu and Tyr. The results suggest that the TK2268 protein is an aminotransferase with specificity toward the acidic amino acids Glu and Asp.

**Table 2:** Kinetic parameters of the aminotransferase reaction catalyzed by the TK2268 protein.

Substrate		*V*_max_ (μmol min^−1^ mg^−1^)	*K*_m_ (mM)	*k*_cat_ (s^−1^)	*k*_cat_/*K*_m_ (mM^−1^ s^−1^)
Amino donor	Amino acceptor				
*Aspartate*	Pyruvate^a^	5.42 ± 0.18	2.14 ± 0.22	4.07 ± 0.14	1.90
*Glutamate*		7.42 ± 0.31	2.17 ± 0.27	5.57 ± 0.23	2.57
*Leucine*		4.68 ± 0.22	36.3 ± 4.3	3.52 ± 0.23	0.10
*Leucine*	2-Oxoglutarate^a^	5.48 ± 0.22	39.6 ± 3.8	4.12 ± 0.23	0.10
*Tyrosine*		0.34 ± 0.02	1.45 ± 0.26	0.26 ± 0.02	0.18
Leucine^b^	*2-Oxoglutarate*	4.45 ± 0.14	0.19 ± 0.03	3.35 ± 0.15	17.6
	*Pyruvate*	3.76 ± 0.15	2.72 ± 0.35	2.83 ± 0.16	1.04

The kinetic parameters are those for the substrates indicated in italic. ^a^Concentration fixed at 10 mM. ^b^Concentration fixed at 50 mM.

### Gene disruption and growth of the ΔTK0548 and ΔTK2268 strains

**Figure 5:**
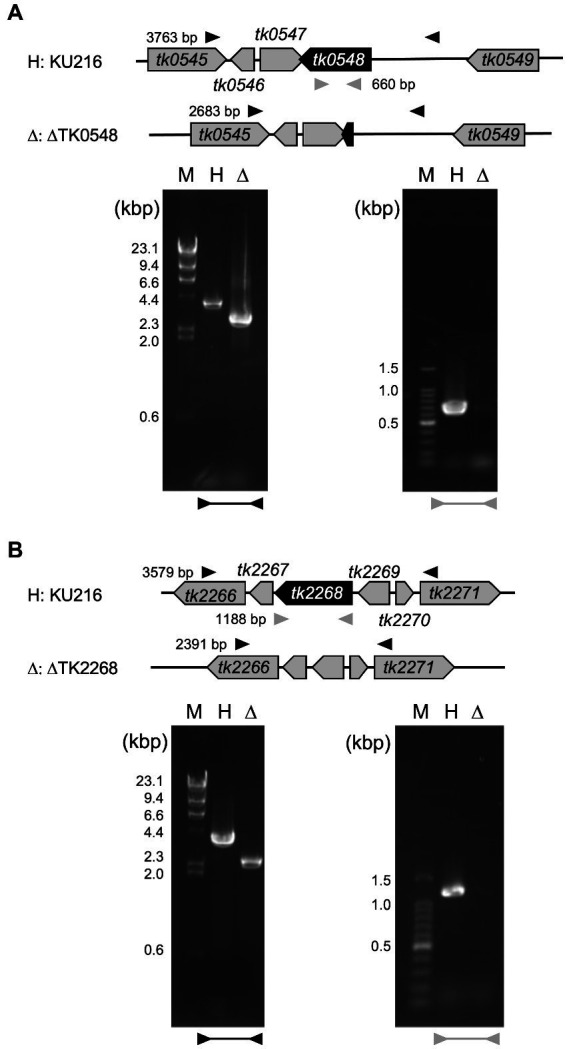
Confirmation of TK0548 **(A)** and TK2268 **(B)** gene disruption by PCR. Predicted gene loci before and after gene disruption are shown above the gels. Arrowheads indicate the position of primers outside the homologous regions used for recombination (black) and inside the coding regions (gray). Amplified fragments were subjected to agarose gel electrophoresis along with DNA markers (M). H, host strain KU216; Δ, gene disruption strains.

To understand the contribution of the TK0548 and TK2268 genes to amino acid catabolism in *T. kodakarensis*, we disrupted each gene using *T. kodakarensis* KU216 (Δ*pyrF*) as a host strain. Five transformants were chosen for each gene disruption and their loci were examined by PCR. Examples of transformants whose TK0548 gene (Figure 5A) or TK2268 gene (Figure 5B) was disrupted are shown. The respective loci were sequenced, confirming that gene disruption had occurred as intended.

**Figure 6:**
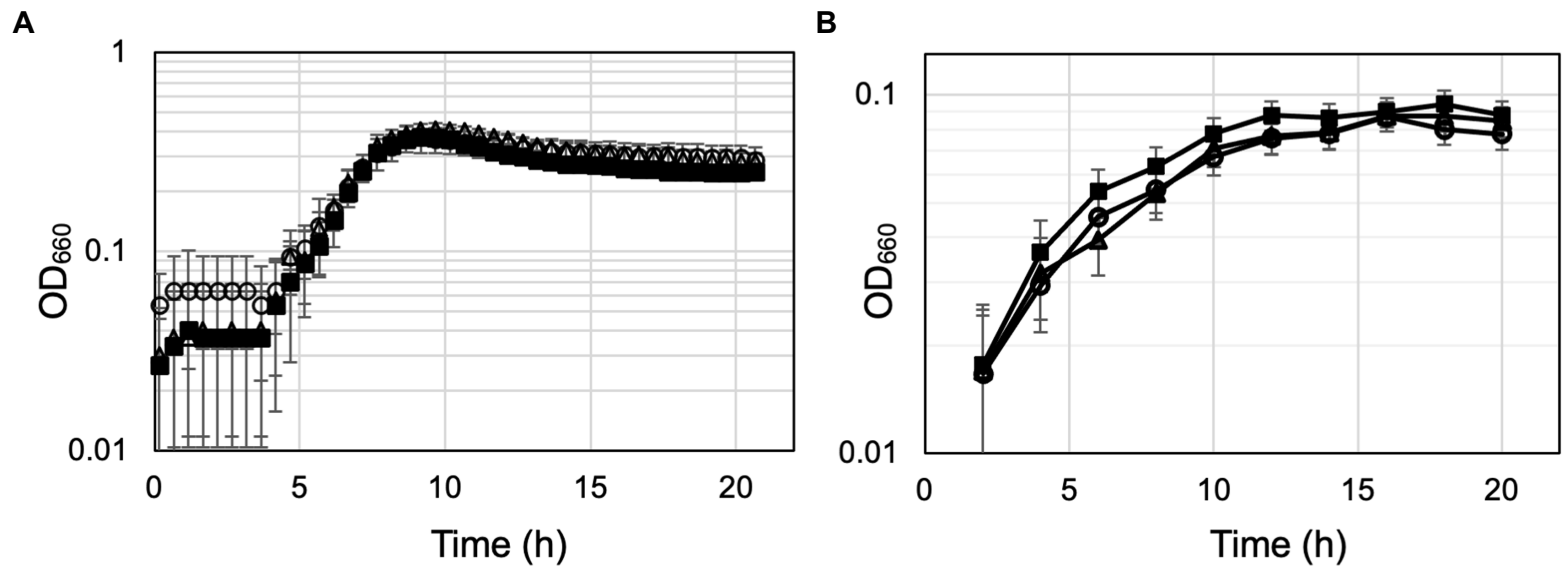
Growth properties of the host strain KU216 and the gene disruption strains. Growth of the host KU216 strain (closed squares) and the ΔTK0548 (open triangles) and ΔTK2248 (open circles) gene disruption strains were examined in a nutrient-rich ASW-YT-m1-S^0^ medium containing yeast extract and tryptone **(A)**, and in a synthetic amino acid medium ASW-AA-m1-S^0^(+Ura) **(B)**. Growth was measured at 85°C. Error bars indicate the standard deviations of three independent culture experiments. The vertical axis is represented in logarithmic scale.

The ΔTK0548 and ΔTK2268 disruption strains were first grown in the nutrient-rich ASW-YT-m1-S^0^ medium containing yeast extract and tryptone (Figure 6A). The growth of both disruption strains did not display significant differences to that of the host strain KU216. Therefore, in order to increase the dependency of growth on amino acid catabolism, the strains were grown in ASW-AA-m1-S^0^(+Ura) medium (Figure 6B). This medium is a synthetic medium with amino acids as the only major carbon and energy source. In this case, we observed a retardation in growth in both gene disruption strains. Cell yields of the cultures eventually reached similar levels, but the gene disruption strains took a 4-h longer period of time to reach maximum cell density. The results suggest that both proteins are involved in the utilization of amino acids for growth of *T. kodakarensis*.

### Aminotransferase activity in *Thermococcus kodakarensis* cell extracts

**Figure 7:**
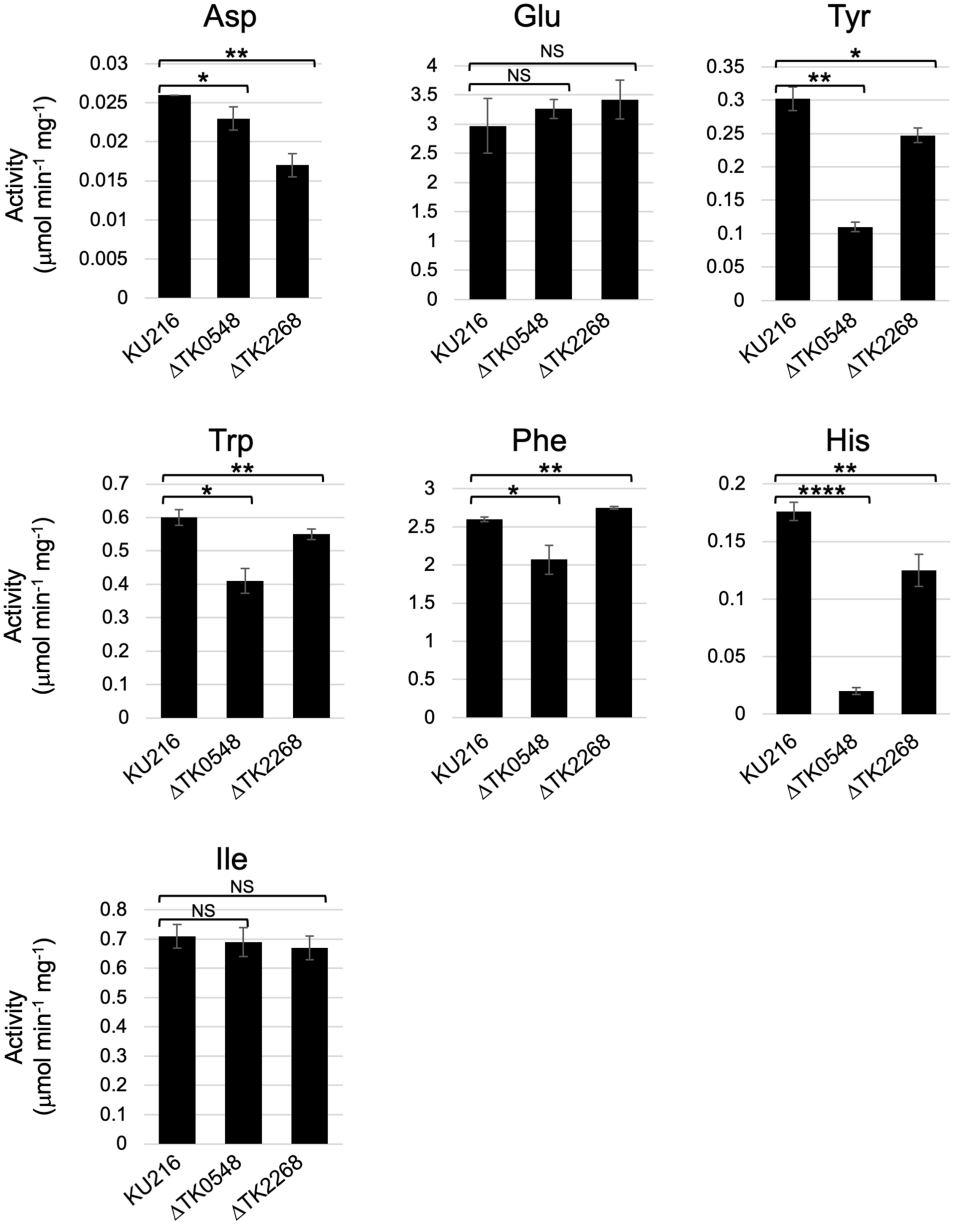
Aminotransferase activity in *T. kodakarensis* cell extracts. Cell extracts were incubated with different amino acids and 2-oxoglutarate, or pyruvate (for Glu and Asp), at 80°C for various periods of time. Activity was calculated based on the generation of Glu or Ala. Amino donors and acceptors were added at a concentration of 10 mM (Tyr: 6 mM). 0.01 < *p* < 0.05*; 0.001 < *p* < 0.01**; 0.0001 < *p* < 0.001***; *p* < 0.0001****; not significant: NS.

The substrate specificities of multiple aminotransferases from members of Thermococcales have been determined *in vitro*. However, the contribution of each protein in the conversion of a particular amino acid *in vivo* cannot be determined by biochemical properties alone, as multiple aminotransferases, in some cases with overlapping substrate specificities or different expression levels, are present in the cell. We thus measured and compared aminotransferase activity in the cell extracts of *T. kodakarensis* host strain and gene disruption strains. As *in vitro* studies indicated that the TK0548 protein preferred Trp, Phe, Tyr, and His, while the TK2268 protein recognized Asp and Glu, activities in the cell extracts toward Trp, Phe, Tyr, His, Asp, and Glu were measured. We also used Ile as an amino donor. *In vitro* studies indicated that the recognition of both TK0548 and TK2268 proteins toward Ile was minimal (Figure 3), so the effects of TK0548 and TK2268 disruption on intracellular aminotransferase activity toward Ile would be expected to be low. As shown in Figure 7, aminotransferase activity for all seven amino acids was clearly observed in KU216 cell extracts. We observed that the levels of aminotransferase activity toward different amino acids greatly differed. Activities toward Phe or Glu were particularly high, whereas that toward Asp was notably low, two orders of magnitude lower than those observed for Phe or Glu. When we examined the effects of gene disruption, the disruption of TK0548 and TK2268 had no effect on intracellular aminotransferase activity toward Ile, consistent with our *in vitro* results that neither enzyme recognized Ile (Figure 3). We next examined differences observed upon gene disruption, focusing on those with *p* values below 0.01. We observed that the disruption of TK2268 resulted in a 35% decrease in aminotransferase activity toward Asp, one of the substrates preferred by the protein in *in vitro* experiments. Disruption of TK2268 did not affect the Glu aminotransferase activity in cell extracts, suggesting that the contribution of the protein to this activity is relatively small. This is not surprising though, as *in vitro* analysis indicated that the activity of the TK2268 protein toward Glu and Asp are comparable (Table 2). As the total Glu aminotransferase activity in *T. kodakarensis* cell extracts is over 100-fold higher than that toward Asp, a decrease in Glu aminotransferase activity comparable to the levels toward Asp would correspond to only a very small fraction of the total activity toward Glu. We also observed a decrease in His aminotransferase activity, but this could not be explained by the results of *in vitro* analysis. When TK0548 was disrupted, decreases in activity toward Trp (32%), Tyr (64%), and His (89%) were observed. The TK0548 protein seems to be a major contributor for Tyr and His aminotransferase activity in *T. kodakarensis*.

## Discussion

Biochemical studies have been carried out on wealth of aminotransferases from Thermococcales species. Proteins from *T. kodakarensis*, *P. furiosus*, *P. horikoshii*, and *T. litoralis* that are classified in the Class I to Class IV aminotransferases are listed in Table 3. Some proteins have been shown to catalyze reactions other than transamination, and include racemases, decarboxylases, desulfurases, hydroxymethyltransferases and others. Concerning the aminotransferases that have been biochemically examined, the amino donor and acceptor molecules are indicated. The TK0548 protein was shown here to prefer the aromatic amino acids Phe, Tyr and Trp, which is consistent with other members of G1. To a lower degree, the protein also recognized His. The PH1371 protein also displays activity toward His (Matsui et al., 2000). The activities of the PF1253 (Ward et al., 2002) and OCC_04737 (Andreotti et al., 1994) proteins toward His have not been examined. In the case of the TK2268 protein, it is the first characterized representative of G2, and displays specificity toward the acidic amino acids Asp and Glu. Although the range of substrates examined in separate studies differ, enzymes that belong to the same group have been shown to display similar substrate specificities, such as the four enzymes from G1 and the three enzymes from G6. Our results with the TK2268 protein thus raise the possibilities that members of G2 recognize acidic amino acids as amino donors.

**Table 3:** An overview of aminotransferase homologs in Thermococcales.

Species	Class	Gene number	Group	Function (Annotation)^*^	Amino donor^**^	Amino acceptor^**^	Reference
*Pyrococcus furiosus*	I	PF1253	G1	Aromatic aminotransferase	Phe, Tyr, Trp	2-OG	Ward et al. (2002)
		PF0522	G2	(Aspartate aminotransferase)			
		PF1497	G3	Alanine aminotransferase	Ala, Glu, (Asp, Ile, Leu)	2-OG, Pyr	Ward et al. (2000)
		PF1702	G4	Aspartate aminotransferase	Asp, (Glu)	2-OG, (Pyr, phenylpyruvate)	Ward et al. (2002)
		PF0121	G6	Aromatic aminotransferase	Phe, Tyr, Trp	2-OG	Andreotti et al. (1995)
		PF1665	G7	(Histidinol-phosphate aminotransferase)			
		PF0293	G8	(Histidinol-phosphate aminotransferase)			
	II	PF1685		(Acetylornithine aminotransferase)			
		PF1421		(4-aminobutyrate aminotransferase)			
		PF1232		(4-Aminobutyrate aminotransferase)			
		PF0513		(Putative glutamate aminotransferase)			
	III	ND		ND			
	IV	PF0164		(Cysteine desulfurase)			
		PF1066		(Putative aminotransferase)			
		PF1472		(Aspartate/serine transaminase)			
		PF1778		(Serine hydroxymethyltransferase)			
		PF1999		(Glycine dehydrogenase subunit 1)			
		PF2000		(Glycine dehydrogenase subunit 2)			
*Pyrococcus horikoshii*	I	PH1371	G1	Aromatic aminotransferase	Phe, Tyr, Trp, (His)	Phenylpyruvate, 2-OG	Matsui et al. (2000)
		PH0771	G2	(Aspartate aminotransferase)			
		PH1322	G3	(Alanine-synthesizing aminotransferase)			
		PH0207	G6	Kynurenine aminotransferase	Kynurenine	2-OG, OAA, 2-oxobutyrate, 2-oxo-4-methylthiobutyrate	Okada et al. (2012)
		PH0377	G8	(Histidinol-phosphate aminotransferase)			
	II	PH1716		(Long hypothetical acetylornithine aminotransferase)			
		PH1501		(Long hypothetical aminotransferase)			
		PH1423		Ornithine δ-aminotransferase	l-Orn, l-Lys, d-Orn, d-Lys, (5-aminovalerate, 6-aminohexanoate, GABA)	2-OG, (Pyr)	Kawakami et al. (2022)
		PH0782		Alanine/serine racemase	Ala, Ser^***^		Kawakami et al. (2018)
		PH0138		Amino acid racemase	Phe, Leu, Met, (Tyr, Ile, Val, Trp, Ala)^***^		Kawakami et al. (2015, 2017)
	III	ND		ND			
	IV	PH0626		(Long hypothetical protein)			
		PH1308		(Long hypothetical serine aminotransferase)			
		PH1654		(Long hypothetical serine hydroxymethyltransferase)			
		PH1994		(Glycine dehydrogenase subunit 2)			
		PH1995		(Glycine dehydrogenase subunit 1)			
*Thermococcus litoralis*	I	OCC_04737	G1	Aromatic aminotransferase	Phe, Tyr, Trp	2-OG	Andreotti et al. (1994)
		OCC_08839	G2	(Aminotransferase)			
		OCC_03788	G3	(Alanine aminotransferase)			
		OCC_03517		(Hypothetical protein)			
		OCC_10965		Aspartate aminotransferase			
		OCC_05516	G4	(Aspartate aminotransferase)			
		OCC_02240	G5	Alanine glyoxylate aminotransferase	Ala	Glyoxylate	Sakuraba et al. (2004)
		OCC_04335	G6	Aromatic aminotransferase	Phe, Tyr, Trp	2-OG	Andreotti et al. (1994)
		OCC_11814		(Histidinol-phosphate aminotransferase)			
		OCC_11984		(Histidinol-phosphate aminotransferase)			
	II	OCC_00582		(4-Aminobutyrate aminotransferase)			
		OCC_08410		(4-Aminobutyrate aminotransferase)			
		OCC_10945		Moderate-substrate specificity amino acid racemase	Met, Leu, (Phe, Ala, Ser)^***^		Kawakami et al. (2021)
	III	ND		ND			
	IV	OCC_11247		(Cysteine desulfurase)			
		OCC_00972		(Aminotransferase)			
		OCC_11879		(Aspartate aminotransferase)			
		OCC_07124		(Glycine dehydrogenase subunit 1)			
		OCC_07119		(Glycine dehydrogenase subunit 2)			
*Thermococus kodakarensis*	I	TK0548	G1	Aromatic aminotransferase	Tyr, Phe, Trp, His	2-OG, (Pyr)	This study
		TK2268	G2	Aspartate aminotransferase	Glu, Asp	2-OG, (Pyr)	This study
		TK1094	G3	(Alanine aminotransferase)			Kanai et al. (2015)
		TK0260	G4	Aspartate aminotransferase			
		TK0186	G6	(2-Aminoadipate transaminase)			
		TK0250	G7	(Histidinol-phosphate aminotransferase)			
		TK0864	G8	(Threonine-*O*-3-phosphate decarboxylase)			
	II	TK0275		LysW-γ-L-lysine aminotransferase			Yoshida et al. (2016)
		TK1211		Leu/Met racemase	Leu, Met^***^		Zheng et al. (2021)
		TK2101		Ornithine ω-aminotransferase	l-Orn, l-Lys, (d-Orn, d-Lys)	2-OG, 2-oxoadipate, (OAA, Pyr)	Zheng et al. (2018)
	III	ND		ND			
	IV	TK1990		Cysteine desulfurase	Cys^***^		Hidese et al. (2014)
		TK1303		(Hypothetical protein, conserved)			
		TK1548		(Probable serine-glyoxylate aminotransferase)			
		TK1379		(Glycine cleavage system protein P, subunit 2)			
		TK1380		(Glycine cleavage system protein P, subunit 1)			

^*^Functions in parentheses are based on annotation. **Compounds in parentheses are less recognized substrates. ***Substrates for the indicated enzymes other than aminotransferases. 2-OG, 2-oxoglutarate; OAA, oxaloacetate; Orn, ornithine; Pyr, pyruvate; GABA, γ-aminobutyric acid; ND, not detected.

In order to understand the contribution of each enzyme to the transamination of a specific amino acid *in vivo*, we examined and compared specific aminotransferase activities among cells of the host strain KU216 and ΔTK0548 and ΔTK2268 disruption strains. This takes into account not only the substrate specificity and activity levels of each enzyme, but also their expression levels in the cell. The results suggested that TK0548 contributed to the aminotransferase activity toward Phe and Trp, and to a higher degree Tyr. The considerable levels of activity still observed in the disruption strains most likely reflects the activity of the TK0186 protein, a member of G6 (Table 3). Interestingly, the TK0548 protein seems to be the predominant His aminotransferase in *T. kodakarensis*, accounting for approximately 90% of the activity in cell extracts.

**Figure 8:**
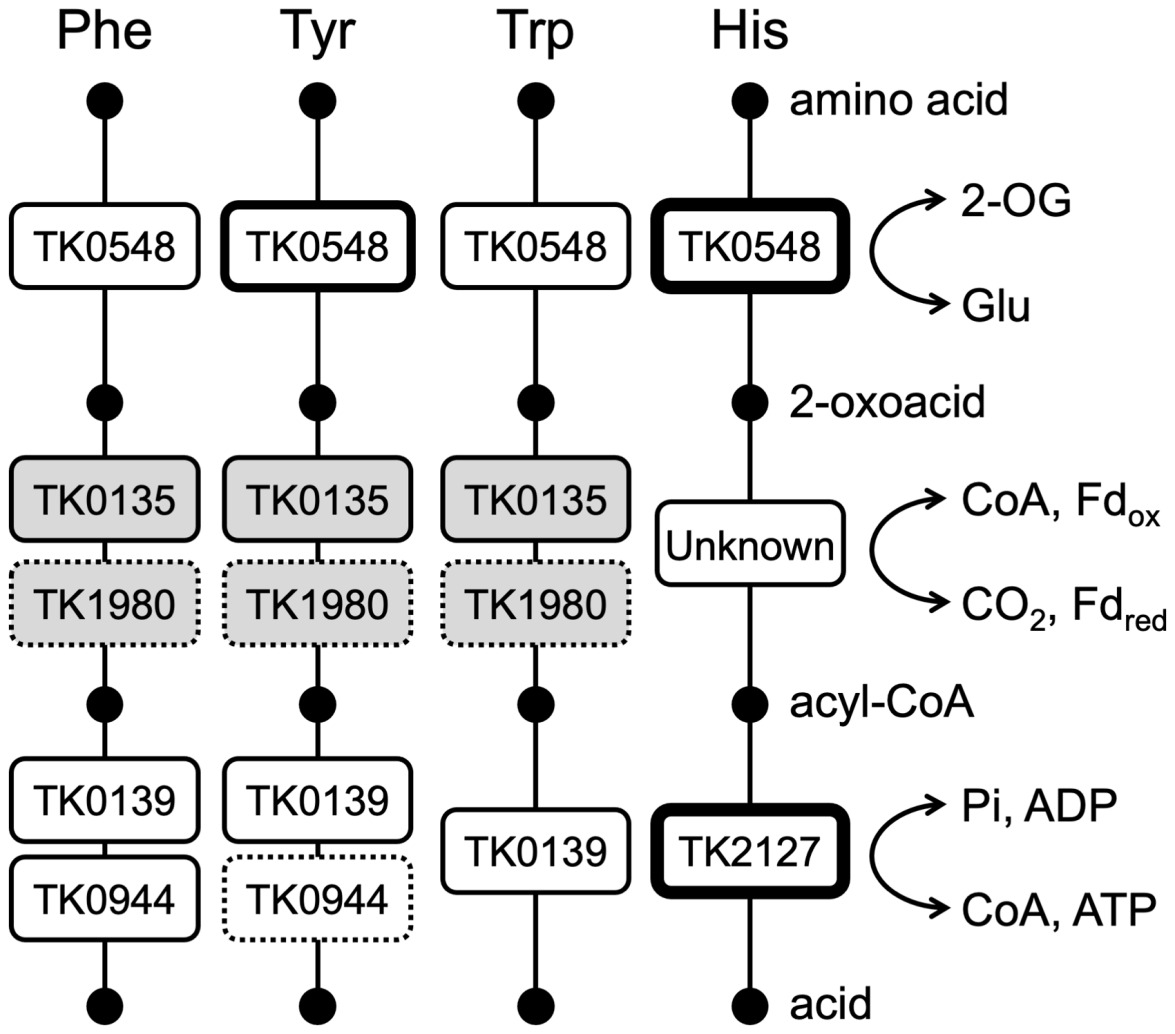
A diagram illustrating the involvement of aminotransferases, 2-oxoacid:ferredoxin oxidoreductases, and acyl-CoA synthetases in the catabolism of Phe, Tyr, Trp, and His in *T. kodakarensis*. Enzymes with greater roles in each step are indicated with boxes with thicker lines, and those with minor roles are indicated by dotted lines. Gene numbers of the acyl-CoA synthetases indicate those of the α subunit. TK0135 represents TK0135-0136, TK1980 represents TK1978-1981. The genes in white boxes are based on biochemical and genetic evidence and the shaded boxes are predicted based on similarity to the experimentally examined enzymes from *P. furiosus*. 2-OG, 2-oxoglutarate; CoA, coenzyme A; Fd, ferredoxin; Pi, phosphate.

Amino acid catabolism in members of the Thermococcales proceeds *via* amino acid, 2-oxoacid, acyl-CoA and acid (Figure 8). Concerning the conversion from amino acids to 2-oxoacids, our group has previously shown that Glu is the only amino acid that is converted by a dehydrogenase, a glutamate dehydrogenase encoded by TK1431. All other amino acids that are converted to 2-oxoacids would have to rely on aminotransferases (Yokooji et al., 2013). The 2-oxoacids are converted to acyl-CoAs *via* 2-oxoacid:ferredoxin oxidoreductases. There are seven potential sets of genes encoding 2-oxoacid:ferredoxin oxidoreductases in members of the Thermococcales, and four protein complexes from *P. furiosus* have been biochemically examined; pyruvate:ferredoxin oxidoreductase (POR), 2-ketoisovalerate:ferredoxin oxidoreductase (VOR), indolepyruvate:ferredoxin oxidoreductase (IOR), and 2-ketoglutarate:ferredoxin oxidoreductase (KGOR) (Blamey and Adams, 1993; Mai and Adams, 1994; Heider et al., 1996; Mai and Adams, 1996). The KGOR homolog in *T. kodakarensis* has been genetically examined, confirming its role in Glu metabolism, converting 2-oxoglutarate to succinyl-CoA (Yokooji et al., 2013). Finally, the acyl-CoAs are hydrolyzed by NDP-forming acyl-CoA synthetases. There are five NDP-forming acyl-CoA synthetases in Thermococcales; ACS I, ACS II, ACS III, succinyl-CoA synthetase (SCS), and 2-(imidazol-4-yl)acetyl-CoA synthetase (ICS). ACS I and ACS II from *P. furiosus* and ACS II, ACS III, SCS and ICS from *T. kodakarensis* have been biochemically examined (Mai and Adams, 1996; Glasemacher et al., 1997; Musfeldt et al., 1999; Shikata et al., 2007; Awano et al., 2014). The involvement of SCS in Glu metabolism has been genetically confirmed (Yokooji et al., 2013). As the NDP-forming acyl-CoA synthetases catalyze the reaction in which substrate-level phosphorylation occurs, it is possible to predict the amino acids that are subject to this catabolism based on the substrate specificities of the acyl-CoA synthetases. The substrates recognized by the five acyl-CoA synthetases combined suggest that the amino acids that are subject to this mode of catabolism are Ala, Val, Ile, Leu, Met, Phe, Tyr, Trp, Glu/Gln, Cys, and His (Awano et al., 2014). Aminotransferases that recognize Ala, Val, Ile, Leu, Met, Phe, Tyr, Trp, and Glu/Gln have been reported, as well as 2-oxoacid:ferredoxin oxidoreductases that act on the corresponding 2-oxoacids after transamination. However, aminotransferases and 2-oxoacid:ferredoxin oxidoreductases that are related to Cys and His degradation are still not known. In particular, ICS displays a remarkably high specificity toward 2-(imidazol-4-yl)-acetate, suggesting that it is involved solely in His metabolism (Awano et al., 2014). The results of this study strongly suggest that the TK0548 protein and ICS are metabolically linked in His catabolism (Figure 8). To a certain extent, the TK0548 protein also contributes in the catabolism of aromatic amino acids, particularly Tyr. Finally, the specificities of the five acyl-CoA synthetases combined raise the possibilities that Asp, Asn, Gly, Lys, Arg, Pro, Ser, and Thr may not be directed to the catabolic degradation involving 2-oxoacid:ferredoxin oxidoreductases and NDP-forming acyl-CoA synthetases, and this may be related to the notably low Asp aminotransferase activity observed in *T. kodakarensis* cell extracts and the TK2268 protein. As we have previously shown that *T. kodakarensis* does not harbor an Asp dehydrogenase, our present knowledge suggests that Asp is not catabolized through the pathway involving 2-oxoacid:ferredoxin oxidoreductases and NDP-forming acyl-CoA synthetases. We also could not find an aspartate ammonia lyase homolog on the genome, which would lead to the generation of fumarate. In addition, *T. kodakarensis* possesses a fumarase homolog, but lacks a number of homologs of the citric acid cycle including succinate dehydrogenase, malate dehydrogenase and citrate synthase. Other than the TK2268 protein, enzymes potentially related to oxaloacetate metabolism are a putative fumarase, malic enzyme (Fukuda et al., 2005), and phosphoenolpyruvate carboxykinase (Fukuda et al., 2004). *T. kodakarensis* may display a C4-compound metabolism distinct to those found in bacteria. Further understanding of oxaloacetate metabolism may provide valuable clues to elucidate the metabolism of Asp and the physiological function of TK2268. We would like to note, however, that we cannot rule out the possibility that the TK2268 protein recognizes a substrate completely different from those considered in this study.

## Materials and methods

### Strains and culture conditions

*Thermoccocus kodakarensis* was isolated from Kodakara Island, Kagoshima, Japan (Morikawa et al., 1994; Atomi et al., 2004). *T. kodakarensis* KU216 (Sato et al., 2003, 2005) and derivative strains were cultivated under strictly anaerobic conditions at 85°C in nutrient-rich medium (ASW-YT-m1-S^0^ or ASW-YT-m1-pyruvate) or synthetic medium (ASW-AA-m1-S^0^). ASW-YT-m1-S^0^, ASW-YT-m1-pyruvate, and ASW-AA-m1-S^0^ are modified versions of ASW-YT-S^0^, ASW-YT-pyruvate, and ASW-AA-S^0^ media, respectively. ASW-YT-S^0^ was composed of 0.8 × artificial seawater (ASW) (Robb and Place, 1995), 5 g L^−1^ yeast extract, 5 g L^−1^ tryptone, and 2 g L^−1^ elemental sulfur. In ASW-YT-m1-S^0^, 20 μM KI, 20 μM H_3_BO_3_, 10 μM NiCl_2_, and 10 μM Na_2_WO_4_ were supplemented. In ASW-YT-m1-pyruvate medium, elemental sulfur was replaced with 5 g L^−1^ sodium pyruvate. ASW-AA-S^0^ was composed of 0.8 × ASW, a mixture of 20 amino acids, modified Wolfe’s trace minerals and a mixture of vitamins (Sato et al., 2003). In ASW-AA-m1-S^0^, 20 μM KI, 20 μM H_3_BO_3_, 10 μM NiCl_2_, and 10 μM Na_2_WO_4_ were supplemented, and the concentrations of l-arginine hydrochloride and l-valine were increased (from 125 mg L^−1^ to 250 mg L^−1^ and from 50 mg L^−1^ to 200 mg L^−1^, respectively). When cells without a *pyrF* gene were grown, 10 μg mL^−1^ uracil was added to make ASW-AA-m1-S^0^(+Ura). To remove oxygen in the medium, 5% (w/v) Na_2_S solution was added until the medium became colorless. Resazurine (0.5 mg L^−1^) was also added to all media as an oxygen indicator. For solid medium used to isolate transformants, 10 g L^−1^ gelrite, 7.5 g L^−1^ 5-fluoroorotic acid (5-FOA), 10 μg mL^−1^ uracil, 4.5 mL of 1 M NaOH and 0.2% (v/v) polysulfide solution (10 g Na_2_S 9H_2_O and 3 g sulfur flowers in 15 mL H_2_O) rather than elemental sulfur was supplemented to ASW-AA-m1 medium. *Escherichia coli* DH5α (Takara Bio, Kusatsu, Japan) and BL21-Codonplus(DE3)-RIL strains (Agilent Technologies, Santa Clara, CA) were cultivated at 37°C in Lysogeny broth (LB) medium supplemented with ampicillin (100 mg L^−1^). *E. coli* DH5α was used for recombinant plasmid construction and *E. coli* BL21-Codonplus (DE3)-RIL was used for heterologous gene expression. Chemicals were purchased from Wako Pure Chemicals (Osaka, Japan) or Nacalai Tesque (Kyoto, Japan) unless mentioned otherwise.

### Expression of the TK0548 and TK2268 genes

In this study, pET21a(+) was used as an expression vector for TK0548 which was amplified from genomic DNA of *T. kodakarensis* KU216 using the primer set TK0548F/TK0548R (Table 4). The restriction enzyme sites *Nde*I and *Bam*HI were incorporated into the 5′- and 3′-termini of the fragments, respectively, during PCR. The amplified product and pET21a(+) were digested with *Nde*I and *Bam*HI, ligated using Ligation high Ver. 2, and introduced into *E. coli* DH5α cells. Positive colonies were selected by PCR analysis and confirmed by DNA sequencing. Plasmids were introduced into *E. coli* BL21-Codonplus (DE3)-RIL for gene expression.

**Table 4:** Primers used in the study.

Primer	Sequence (5′ to 3′)	Use
TK0548F	AAAAACATATGGCGCTGAGCGACAGGCTTGACC	Construction of expression plasmids
TK0548R	AAAGGATCCTTAAACGAGCTTTTTCTCCTTCAGG	
TK2268F	AAAAAAACATATGAGGTATAAGAAGAGAAAGTAC	
TK2268R	AAAGTCGACTCAGTGGTGGTGGTGGTGGTGCAGCTTCGAGAGGGCCT	
TK0548seqF1	TCAGCGAGCTTATGCTCAAG	Sequencing
TK0548seqR1	GCATTGGAAGGCCCGCGTTCGG	
TK2268seqF1	CCCCAACGCAGGCATCC	
TK2268seqF2	CCAAAATAGCCGAGGCAGGG	
TK2268seqF3	TACATCCTCAGCGACGAGCCC	
TK2268seqF4	CGGCAACGTTACGTCCTTCATCC	
TK2268seqF5	TCGATATGACCAGCGAAGAC	
TK2268seqR1	CCCTGCCTCGGCTATTTTGG	
dTK0548seqF1	GGCAGCCCTGAGTGAGGGCGTTG	
dTK0548seqF2	GGCGGTGTTGATGCGTTCTTC	
dTK0548seqF3	ATTGAAATTCCGCAGAGCCATTG	
dTK0548seqF4	CCGCGTCAACTTCGAGGCTCTC	
dTK0548seqF5	GTGATACAATATGGAGAAGG	
dTK0548seqR1	CATAATCCTCTCCCACGCTCCTCCGAAG	
dTK0548seqR2	CCGGGGACATAACGAACTTC	
dTK0548seqR3	CCTTCTCCATATTGTATCAC	
dTK0548seqR4	CCTCACCACTCCCCAAAGTC	
TK0548seqF3	ATGGTAATTAGCGATGAAGTTTACG	
TK0548seqF4	CCTGTCCGGTCACCTTCGC	
TK0548seqF5	TCAGCGAGCTTATGCTCAAG	
TK0548seqR2	GAACGCCTGGTTGGCTCCGGTTAGG	
TK0548seqF6	CCGAACGCGGGCCTTCCAATGC	
TK0548seqF2	AAAGCCCGTTGAGGTTCC	
dTK2268seqF1	GAATGACCTCACTCCTGTTC	
dTK2268seqF2	CTGAACGATGAGACAGGTGATTG	
dTK2268seqF3	CCCACCAGATAAGGGAAGCAATAAAAG	
dTK2268seqF4	TTGTCGCAGAAAGACCCTTCGCCAAG	
dTK2268seqR1	ATCCCCCCTCACATTTTTCC	
dTK2268seqR2	AGTGCTATCGCGACGCTCCTC	
dTK2268seqR3	TTCCAGCCAACCGCTTCGAC	
dTK2268seqR4	CTCCCGCAGGGGCTAATAAACG	
dTK0548F1	GAGAATTGAAACAAAAAGGTGGGTGGTG	Construction of disruption plasmids
dTK0548R1	GAGATCAGACTGCGGGAGCGCTCTGCTTG	
dTK0548F2	ATCAGGATAAGCTACGCCACGGCCTAC	
dTK0548R2	TACCATCACCGCTCTCCCTTC	
dTK0548inF	CGCTGGGATGCAGGATGTTATC	
dTK0548inR	GTAGCCTTCTCCGGCTTTC	
dTK0548outF	ATTGAAACTATAGGGAAATATAGGG	
dTK0548outR	TAATCCCGAAGAAGGGCTACTC	
dTK2268F1	ATCGTCGTCATCGAACTGAG	
dTK2268R1	TGGAGACCTGGTCTTTTGTCC	
dTK2268F2	ATGGAAAACCTGCTTGCCGTCTTCGTTTC	
dTK2268R2	CGATGTTCCCCCCGGGCAGTTCGGGAATG	
dTK2268inF	ATGAGGTATAAGAAGAGAAAGTACTTCATGGCCGGCAGGATAA	
dTK2268inR	GGAACCACCTCACAGCTTCGAGAGGGCCTCTTTCATTC	
dTK2268outF	GGATCGCCGCCAGAACCCTTTC	
dTK2268outR	CATGGCATAGTTCCCTGCAAGCATTG	

The TK2268 gene was amplified from the genomic DNA of *T. kodakarensis* KU216 using the primer set TK2268F/R (Table 4). *Nde*I and *Sal*I sites as well as a sequence to introduce a C-terminal His_6_-tag were incorporated during amplification. The amplified product was digested with *Nde*I and *Sal*I and inserted into a *T. kodakarensis*-*E. coli* shuttle plasmid previously used for heterologous expression of TK2101 and TK1211 (Zheng et al., 2018, 2021). After confirming the absence of unintended mutations by DNA sequencing, the plasmid was introduced into *T. kodakarensis* KPD2 (Δ*pyrF*, Δ*pdaD*, Δ*chiA*) for gene expression. *pdaD* corresponds to TK0149, and disruption of this gene results in agmatine auxotrophy (Fukuda et al., 2008). For transformation, *T. kodakarensis* KPD2 was grown in ASW-YT-m1-S^0^ medium supplemented with agmatine (1.0 mM) at 85°C for 12 h. Cells were harvested by centrifugation (12,000 × *g*, 5 min, 4°C), and resuspended in 200 μL 0.8 × ASW-m1, followed by incubation on ice for 30 min. After mixing with 3.0 μg of the expression plasmid, the mixture was further incubated on ice for 1 h. Cells were inoculated into 20 mL ASW-YT-m1-S^0^ medium. After incubation at 85°C for 24 h, a 200-μL aliquot was further inoculated into 20 mL ASW-YT-m1-S^0^ medium. After incubation at 85°C 24 h, cells were spread onto solid ASW-YT-m1-S^0^ medium. After incubation at 85°C for 24 h, transformants displaying agmatine prototrophy were isolated and cultivated in ASW-YT-m1-S^0^. The presence of recombinant plasmids and absence of unintended mutation were confirmed by PCR and DNA sequencing, respectively.

### Purification of the recombinant proteins

Transformants with TK0548 expression plasmid were cultivated in LB medium (100 mg L^−1^ ampicillin and 30 mg L^−1^ chloramphenicol) at 37°C until the OD_660_ reached 0.6. Heterologous gene expression was induced by adding isopropyl-1-thio-β-D-galactopyranoside (IPTG) to a final concentration 0.1 mM followed by cultivation at 18°C for 20 h. Cells were harvested *via* centrifugation (12,000 × *g*, 15 min, 4°C), and resuspended in 50 mM HEPES buffer (containing 150 mM NaCl, pH 7.5). Sonication was used to lyse the cells and the insoluble cell debris was separated by centrifugation at 12,000 × *g* for 15 min at 4°C. The soluble cell extract was heat treated at 85°C for 15 min, and the thermolabile proteins derived from the host were removed by centrifugation (12,000 × *g*, 15 min, 4°C). The supernatant was loaded onto an anion exchange column (Resource Q) equilibrated with 50 mM HEPES buffer (pH 7.5). Protein was eluted with a linear gradient of 0 to 1.0 M NaCl. Fractions that contain TK0548 protein were collected and concentrated with an Amicon Ultra centrifugal filter unit (MWCO 10000). The resulting protein solution was applied to a Superdex 200 10/300 Gl gel-filtration column (GE Healthcare) with a mobile phase of 50 mM HEPES buffer (containing 150 mM NaCl, pH7.5) at a flow rate of 0.7 mL min^−1^.

The TK2268 gene expression strain was cultivated in ASW-YT-m1-pyruvate medium at 85°C for 20 h, and cells were collected by centrifugation (6,000 × *g*, 15 min, 4°C). After washing with 0.8 × ASW-m1, cells were resuspended in binding buffer (50 mM HEPES buffer, 20 mM imidazole, 500 mM KCl, 10% (v/v) glycerol, pH 7.5), then disrupted by sonication. Insoluble cell debris was removed by centrifugation (12,000 × *g*, 15 min, 4°C). The soluble cell extract was applied to a His GraviTrap column (GE Healthcare) which had been equilibrated with binding buffer. The TK2268 protein with a His_6_-tag at its C terminus was eluted by elution buffer (50 mM HEPES buffer, 500 mM imidazole, 500 mM KCl, 10% (v/v) glycerol, pH 7.5). After concentrating the eluate, it was applied to a Superdex 200 10/300 Gl gel-filtration column (GE Healthcare). The TK2268 protein was eluted with a mobile phase of 50 mM HEPES buffer (pH7.5) containing 500 mM KCl and 10% (v/v) glycerol, at a flow rate of 0.7 mL min^−1^.

For examining the molecular mass of proteins, Blue 2000 was used to examine the void volume of the column, and ribonuclease A (13.7 kDa), carbonic anhydrase (29 kDa), ovalbumin (44 kDa), conalbumin (75 kDa), aldolase (158 kDa), and ferritin (440 kDa; GE Healthcare) were used as standards. The mobile phase was 50 mM HEPES buffer (pH7.5) containing 500 mM KCl, 10% (v/v) glycerol, and the flow rate was 0.7 mL min^−1^. Protein concentrations were determined with a Protein Assay kit (Bio-Rad, Hercules, CA) using bovine serum albumin as standard.

### Construction of gene disruption strains ΔTK0548 and ΔTK2268

Gene disruption strains were constructed using *T. kodakarensis* KU216 (Δ*pyrF*), which shows uracil auxotrophy, as a host strain. For disrupting the TK0548 gene, the region from the start codon to base number 1080 of TK0548 gene was deleted instead of the stop codon to avoid disturbing expression of the overlapping downstream gene. In the case of TK2268, the entire coding region, along with 9 bases of its 3′-flanking region, was deleted. The TK0548 and TK2268 genes along with their 5′- and 3′-flanking regions (~1.0 kbp) were amplified from the genome of *T. kodakarensis* KU216 using the primer sets dTK0548F1/R1 and dTK2268F1/R1 (Table 4). The amplified products were inserted in the *Hinc*II site of the plasmid pUD3 which contains the *pyrF* gene of *T. kodakarensis* inserted in the *Apa*I site of pUC118 (Yokooji et al., 2009). Inverse PCR was performed with the primer sets dTK0548F2/0548R2 and dTK2268F2/2268R2 (Table 4) to remove sequences from the recombinant plasmid. The sequences of relevant regions were confirmed by DNA sequencing.

*Thermococcus kodakarensis* KU216 was cultivated in ASW-YT-m1-S^0^ medium for 12 h. Cells were harvested and resuspended in 200 μL of 0.8 × ASW-m1, then kept on ice for 30 min. After addition of 3.0 μg of the disruption plasmid and further incubation on ice for 1 h, cells were cultivated in ASW-AA-m1-S^0^ medium without uracil for 48 h at 85°C. A 200 μL aliquot was inoculated into fresh ASW-AA-m1-S^0^ medium and further cultivated under the same conditions to enrich transformants displaying uracil prototrophy. The culture was spread onto ASW-YT-m1 solid medium supplemented with 7.5 g L^−1^ 5-FOA and 60 mM NaOH. Only cells that have undergone a pop-out recombination can grow in the presence of 5-FOA. After cultivation at 85°C for 48 h, colonies were selected, and their genotypes were analyzed by PCR using primer sets dTK0548outF/0548outR and dTK2268outF/2268outR (Table 4). Transformants that led to amplification of DNA products with the expected size were chosen and cultivated in ASW-YT-m1-S^0^ medium. Gene disruption was also confirmed by DNA sequencing.

### Enzyme activity measurements

Initial examination of the aminotransferase activity of TK0548 and TK2268 proteins were carried out with Glu and pyruvate as amino donor and amino acceptor, respectively. Aminotransferase activity was measured at 80°C unless mentioned otherwise. The standard reaction mixture of TK0548 protein contained 20 μM PLP, 10 mM Glu, 10 mM pyruvate, 6.24 mM NaCl, and 0.4 μg mL^−1^ recombinant protein in 50 mM HEPES buffer (pH7.4). The standard reaction mixture of TK2268 protein contained 20 μM PLP, 10 mM Glu, 10 mM pyruvate, 8.8 mM KCl, 0.18% (v/v) glycerol and 4 μg mL^−1^ recombinant protein in 50 mM HEPES buffer (pH7.4). When the PLP-dependency of activity was measured, PLP was omitted and activity with or without 10 mM hydroxylamine was measured. When PLP was omitted without addition of hydroxylamine, the amino donor and acceptor were 10 mM Leu and 10 mM 2-oxoglutarate, respectively. In the presence of hydroxylamine, the TK0548 protein reaction was measured with Phe and 2-oxoglutarate, while the TK2268 protein reaction was measured with Asp and pyruvate. The reaction mixture without amino donor and amino acceptor was pre-incubated at 80°C for 2 min, and the amino donor and acceptor were added to start the reaction. After further incubation at 80°C for 5 or 15 min, the reaction was stopped through cooling the reaction mixture on ice for 10 min. Proteins were removed with an Amicon Ultra-0.5 centrifugal filter unit with an Ultracel-10 membrane (Millipore). The formation of Glu and Ala was detected and quantified by HPLC after derivatization. The derivatization mixture (100 μL) contained 10 μL of reaction mixture, 70 μL of solution B [borate sodium hydroxide buffer (0.4 M, pH 10.4)] and 20 μL of solution A (8 mg *o*-phthalaldehyde and 10 mg *N*-acetylcysteine were dissolved in 1 mL methanol). After derivatization for 5 min at room temperature, an aliquot (10 μL) of the solution was applied to a COSMOSIL 5C18-PAQ packed column (4.6ID × 250 mm) using a Nexera X2 liquid chromatography system with a fluorescence detector RF-20A XS (Shimadzu, Kyoto, Japan). Compounds were eluted with a solution of 20 mM sodium acetate (pH 5.6) and methanol at a flow rate of 0.7 mL min^−1^. The excitation and emission wavelength were 350 and 450 nm, respectively.

To screen the amino acids recognized by TK0548 and TK2268 proteins, 20 amino acids were used as amino donor, and 2-oxoglutarate or pyruvate was used as amino acceptor. To analyze the aminotransferase activity of TK0548 protein, Tyr, Phe, Trp, His, Leu, Met, and Glu were chosen as amino donor, and 2-oxoglutarate or pyruvate was used as amino acceptor. For the TK2268 protein, Glu, Asp, Cys, Leu, Ala, Met, and Tyr were chosen as amino donor, and 2-oxoglutarate or pyruvate was used as amino acceptor. The standard reaction mixture was incubated at 80°C for 3, 5, and 7 min (reaction mixture with His and Glu were incubated at 80°C for 1, 2, 3 min) to confirm that product formation was linear with time.

For kinetic analysis of the TK0548 protein reaction, reaction rates with various concentrations of Phe, Trp, Tyr, Met, and His were examined with 10 mM 2-oxoglutarate. Reaction rates with various concentrations of 2-oxoglutarate and pyruvate were examined with 10 mM Phe. For analysis of the TK2268 protein, reaction rates with various concentrations of Asp, Glu, Tyr, and Leu were examined with 10 mM pyruvate or 10 mM 2-oxoglutarate. Reaction rates with various concentrations of 2-oxoglutarate and pyruvate were examined with 10 mM Leu. Kinetic parameters were obtained by fitting the data to the Michaelis–Menten equation using IGORPRO, version 6.03 (Wave-Metrics, Lake, Oswego, OR).

### Growth measurements

Growth properties of the host strain KU216 and the ΔTK0548 and ΔTK2268 gene disruption strains were examined in ASW-YT-m1-S^0^ and ASW-AA-m1-S^0^(+Ura) media. Cells were precultured in the nutrient-rich medium ASW-YT-m1-S^0^ for 15 h until the stationary phase and inoculated into ASW-YT-m1-S^0^ medium or synthetic medium ASW-AA-m1-S^0^(+Ura). The OD_660_ of the culture was monitored.

### Activity measurements in cell-free extracts

*Thermococcus kodakarensis* KU216, ΔTK0548 and ΔTK2268 disruption strains were cultivated at ASW-YT-m1-pyruvate medium for 20 h and cells were collected by centrifugation (6,000 × *g*, 15 min, 4°C). Cells were disrupted by sonication and insoluble cell debris was removed (12,000 × *g*, 30 min, 4°C). After exchanging the buffer with 50 mM HEPES (containing 150 mM NaCl, pH7.4) using Amicon Ultra centrifugal filter unit (MWCO 10000), the aminotransferase activity in the cell-free extract was measured. For the aminotransferase activity toward His, Tyr, and Asp, the reaction mixture contained 20 μM PLP, 10 mM amino donor (the final concentration of Tyr was 6 mM), 10 mM amino acceptor (2-oxoglutarate or pyruvate), 5.76 mM NaCl and 0.384 mg mL^−1^ cell-free extracts in 50 mM HEPES buffer (pH7.4). For Trp, the reaction mixture contained 20 μM PLP, 10 mM amino donor, 10 mM amino acceptor (2-oxoglutarate), 2.88 mM NaCl and 0.182 mg mL^−1^ cell-free extracts in 50 mM HEPES buffer (pH7.4). In the case of Glu, Ile, and Phe, the reaction mixture contained 20 μM PLP, 10 mM amino donor, 10 mM amino acceptor (2-oxoglutarate or pyruvate), 0.576 mM NaCl and 0.0384 mg mL^−1^ cell-free extracts in 50 mM HEPES buffer (pH7.4). The reaction mixture without amino acceptor was pre-incubated at 80°C for 2 min, and the amino acceptor was added to start the reaction. After further incubation at 80°C for 1, 2, and 3 min (When Asp and Ile were used as amino donor, the reaction mixtures were incubated at 80°C for 4, 6, and 8 min and 2, 4, and 6 min, respectively). The reaction was stopped through cooling the reaction mixture on ice for 10 min. Proteins were removed with an Amicon Ultra-0.5 centrifugal filter unit with an Ultracel-10 membrane (Millipore). The formation of Glu and Ala was determined by HPLC after derivatization.

## Data availability statement

The original contributions presented in the study are included in the article/Supplementary material, further inquiries can be directed to the corresponding author.

## Author contributions

HA designed the experiments. YS carried out the biochemical and genetic experiments. YM carried out the bioinformatic analyses. All authors contributed in data analyses, writing the manuscript, and approved the submitted version.

## Funding

This study was partially supported by JSPS KAKENHI grant numbers JP19H05679 (Post-Koch Ecology) and JP19H05684 to HA. This work was partially supported by JST SPRING, Grant Number JPMJSP2110 and JST, the establishment of university fellowships toward the creation of science technology innovation, Grant Number JPMJFS2123.

## Conflict of interest

The authors declare that the research was conducted in the absence of any commercial or financial relationships that could be construed as a potential conflict of interest.

## Publisher’s note

All claims expressed in this article are solely those of the authors and do not necessarily represent those of their affiliated organizations, or those of the publisher, the editors and the reviewers. Any product that may be evaluated in this article, or claim that may be made by its manufacturer, is not guaranteed or endorsed by the publisher.

## Supplementary material

The Supplementary material for this article can be found online at: https://www.frontiersin.org/articles/10.3389/fmicb.2023.1126218/full#supplementary-material
