# Supplementary material for: Biochemical and genetic examination of two aminotransferases from the hyperthermophilic archaeon Thermococcus kodakarensis
Source: Front Microbiol. 2023 Feb 20;14:1126218. doi: 10.3389/fmicb.2023.1126218 (PMC9986279; doi:10.3389/fmicb.2023.1126218)
Supplement: Supplementary file 1 [file Data_Sheet_1.PDF]

Table S1. Occurrence of TK0250 and His biosynthesis gene homologs in Thermococcales species.

Organism	TK0250 homolog	His biosynthesis gene cluster
Pyrococcus furiosus DSM 3638	PF1665	
Pyrococcus furiosus COM1	PFC_10280	
Pyrococcus horikoshii		
Pyrococcus abyssi		
Pyrococcus sp. NA2		
Pyrococcus yayanosii		
Pyrococcus sp. ST04	Py04_1649	
Pyrococcus kukulkanii		
Pyrococcus chitonophagus	CHITON_1999	
Thermococcus kodakarensis	TK0250	
Thermococcus onnurineus	TON_0886	
Thermococcus gammatolerans	TGAM_1614	
Thermococcus sibiricus		
Thermococcus barophilus	TERMP_00432	
Thermococcus sp. 4557		
Thermococcus sp. AM4	TAM4_1525	
Thermococcus cleftensis	CL1_1821	
Thermococcus litoralis		
Thermococcus paralvinellae	TES1_0513	
Thermococcus nautili		
Thermococcus eurythermalis		
Thermococcus guaymasensis		
Thermococcus sp. 2319x1		
Thermococcus peptonophilus		
Thermococcus piezophilus	A7C91_10615	
Thermococcus gorgonarius		
Thermococcus celer	A3L02_02960	
Thermococcus barossii	A3L01_05120	
Thermococcus sp. 5-4	CDI07_05060	
Thermococcus siculi		
Thermococcus thioreducens	A3L14_07455	
Thermococcus profundus		
Thermococcus radiotolerans	A3L10_01525	
Thermococcus pacificus		
Thermococcus sp. P6		
Thermococcus indicus		
Thermococcus camini		
Thermococcus aciditolerans	FPV09_02780	
Thermococcus sp. IOH2	K1720_06185	
Palaeococcus pacificus	PAP_03885	

The presence of homologs or predicted gene clusters is indicated in black.

Table S2. Occurrence of TK0260 and Phe/Tyr biosynthesis gene homologs in Thermococcales species.

Organism	TK0260 homolog	Chorismate mutase	Prephenate dehydrogenase	Prephenate dehydratase
Pyrococcus furiosus DSM 3638	PF1702	PF1701	PF1703<br>PF1704	PF0291
Pyrococcus furiosus COM1	PFC_10095	PFC_10100	PFC_10085<br>PFC_10090	PFC_00520
Pyrococcus horikoshii				
Pyrococcus abyssi				
Pyrococcus sp. NA2				
Pyrococcus yayanosii				
Pyrococcus sp. ST04	Py04_1613	Py04_1614	Py04_1612	Py04_0178
Pyrococcus kukulkanii				
Pyrococcus chitonophagus	CHITON_1956	CHITON_1957	CHITON_1955	CHITON_0241
Thermococcus kodakarensis	TK0260	TK0261	TK0259	
Thermococcus onnurineus	TON_1141	TON_1140	TON_1142	
Thermococcus gammatolerans	TGAM_1590	TGAM_1591	TGAM_1589	
Thermococcus sibiricus				
Thermococcus barophilus	TERMP_00428	TERMP_00427	TERMP_00429	
Thermococcus sp. 4557				
Thermococcus sp. AM4				
Thermococcus cleftensis				
Thermococcus litoralis	OCC_05516	OCC_05521	OCC_14085<br>OCC_14090	
Thermococcus paralvinellae	TES1_0491	TES1_0490	TES1_0492	
Thermococcus nautili				
Thermococcus eurythermalis				
Thermococcus guaymasensis				
Thermococcus sp. 2319x1	ADU37_CDS10220	ADU37_CDS10210	ADU37_CDS10230	
Thermococcus peptonophilus				
Thermococcus piezophilus				
Thermococcus gorgonarius				
Thermococcus celer	A3L02_04745	A3L02_04740	A3L02_04750	
Thermococcus barossii				
Thermococcus sp. 5-4				
Thermococcus siculi				
Thermococcus thioreducens				
Thermococcus profundus				
Thermococcus radiotolerans				
Thermococcus pacificus				
Thermococcus sp. P6				
Thermococcus indicus				
Thermococcus camini				
Thermococcus aciditolerans				
Thermococcus sp. IOH2				
Palaeococcus pacificus				

The presence of homologs is indicated in black.

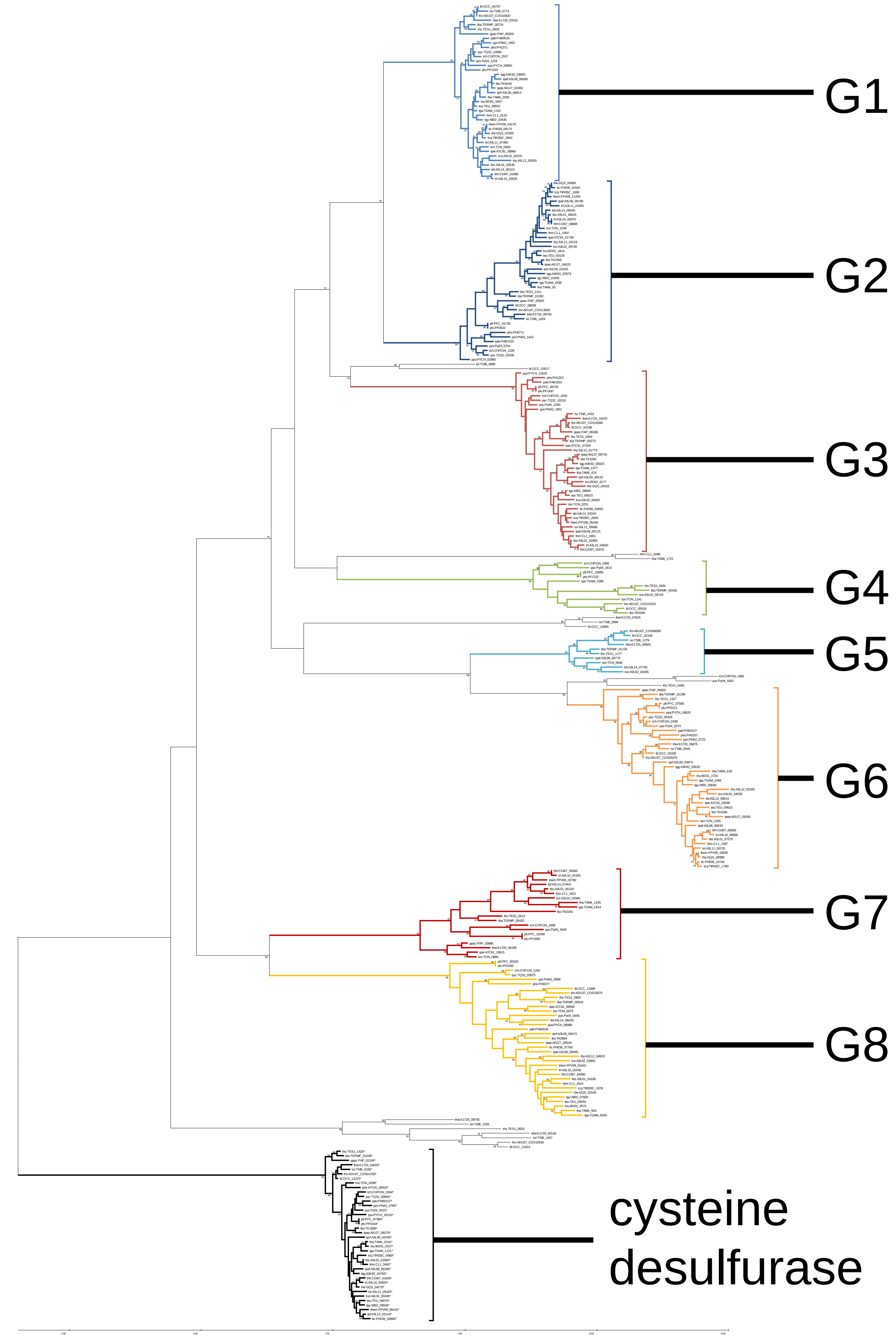
G1
G2
G3
G4
G5
G6
G7
G8
cysteine desulfurase

Fig. S1. Amino acid sequences of class I aminotransferases were collected from all Thermococcales genomes registered on the KEGG database (1-3). Homologs of cysteine desulfarase (TK1990) were added into the dataset as an outgroup. The sequences were aligned by using MUSCLE algorithm (4). The phylogenetic analysis was performed by using the Maximum Likelihood method and JTT matrix-based model (5). The tree with the highest log likelihood (-39302.18) is shown. The percentage of trees in which the associated taxa clustered together is shown next to the branches. Bootstrap values above 50 are shown. Initial tree(s) for the heuristic search were obtained automatically by applying Neighbor-Join and BioNJ algorithms to a matrix of pairwise distances estimated using the JTT model, and then selecting the topology with superior log likelihood value. The tree is drawn to scale, with branch lengths measured in the number of substitutions per site. This analysis involved 291 amino acid sequences. All positions with less than 95% site coverage were eliminated, i.e., fewer than 5% alignment gaps, missing data, and ambiguous bases were allowed at any position (partial deletion option). There were a total of 279 positions in the final dataset. Evolutionary analyses were conducted in MEGA11 (6).

1. Kanehisa, M. and Goto, S.; KEGG: Kyoto Encyclopedia of Genes and Genomes. Nucleic Acids Res. 28, 27-30 (2000).
2. Kanehisa, M; Toward understanding the origin and evolution of cellular organisms. Protein Sci. 28, 1947-1951 (2019).
3. Kanehisa, M., Furumichi, M., Sato, Y., Ishiguro-Watanabe, M., and Tanabe, M.; KEGG: integrating viruses and cellular organisms. Nucleic Acids Res. 49, D545-D551 (2021).
4. Edgar, Robert C. (2004), MUSCLE: multiple sequence alignment with high accuracy and high throughput, Nucleic Acids Research 32(5), 1792-1797.
5. Jones D.T., Taylor W.R., and Thornton J.M. (1992). The rapid generation of mutation data matrices from protein sequences. Computer Applications in the Biosciences 8: 275-282.
6. Tamura K., Stecher G., and Kumar S. (2021). MEGA 11: Molecular Evolutionary Genetics Analysis Version 11. Molecular Biology and Evolution. https://doi.org/10.1093/molbev/msab120.

Fig. S2

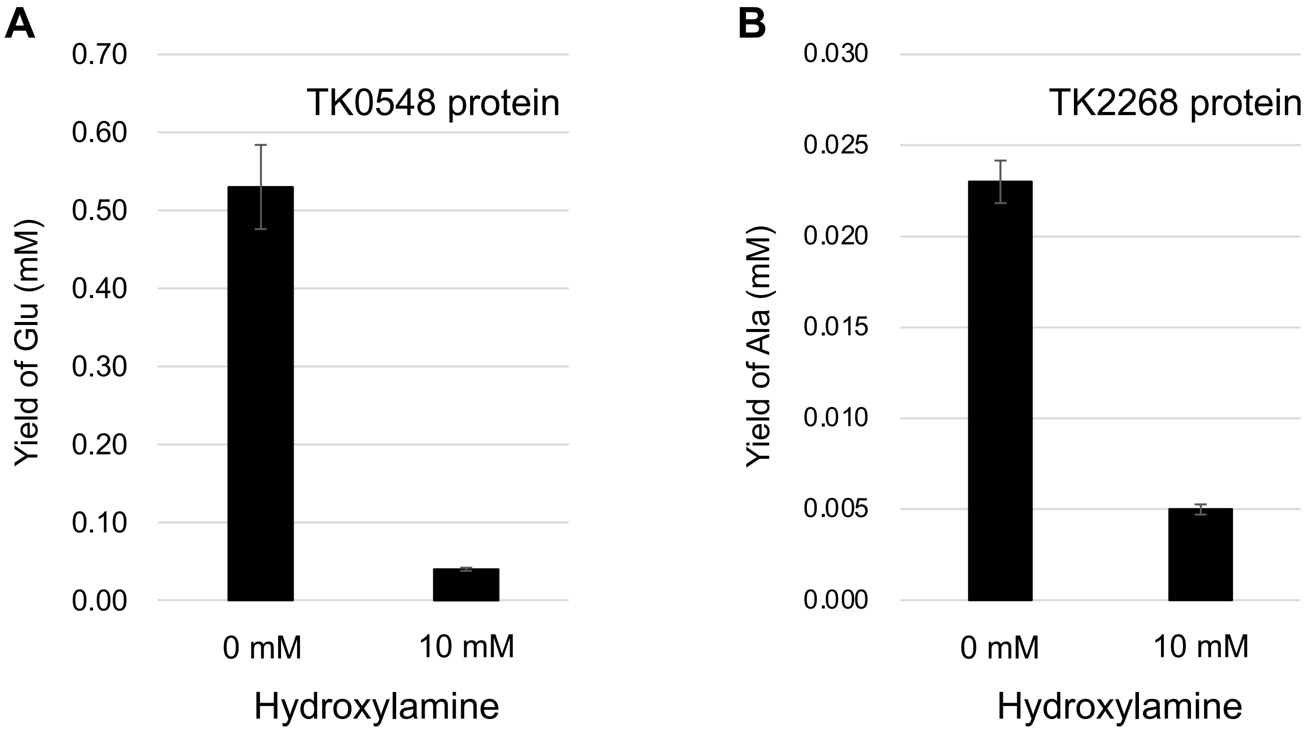

Fig. S2. PLP-dependency of the aminotransferase activity of the TK0548 protein (A) and TK2268 protein (B). Aminotransferase activity was measured in the absence or presence of 10 mM hydroxylamine without the addition of exogenous PLP. The TK0548 protein reaction was measured with 10 mM Phe and 10 mM 2-oxoglutarate, while the TK2268 protein reaction was measured with 10 mM Asp and 10 mM pyruvate. Reactions were carried out for 5 min at 80˚C after treating the proteins with 10 mM hydroxylamine on ice for 1 h. The results are the means of three independent assays and error bars indicate standard deviations.
